# Long-Term Follow-Up After Testicular Tissue Cryopreservation in Boys Facing Gonadotoxic Therapies: Safety, Pubertal Development, Reproductive Outcomes, and Clinical Implications

**DOI:** 10.3390/jcm15197759

**Published:** 2026-10-08

**Authors:** Lucia Paolini, Vittorio Ferrari, Umberto Cattini, Elisa Bortolamedi, Marcello Lanari, Federico Baronio

**Affiliations:** 1 Specialty School of Pediatrics, Alma Mater Studiorum, University of Bologna, 40138 Bologna, Italy; lucia.paolini2@studio.unibo.it; 2 Pediatric Unit, IRCCS Azienda Ospedaliero-Universitaria di Bologna, 40139 Bologna, Italy; umberto.cattini@aosp.bo.it (U.C.); elisa.bortolamedi@aosp.bo.it (E.B.); marcello.lanari@unibo.it (M.L.); federico.baronio@aosp.bo.it (F.B.); 3 Department of Medical and Surgical Sciences, Alma Mater Studiorum, University of Bologna, 40126 Bologna, Italy

**Keywords:** fertility, testicular tissue biopsy, testicular tissue cryopreservation, prepubertal boys, long-term follow-up, gonadotoxic treatment

## Abstract

Testicular tissue cryopreservation is increasingly offered to boys facing gonadotoxic therapies. Yet, its long-term clinical significance depends not only on future fertility-restoration technologies but also on how these patients evolve. This narrative review aims to synthesizes the available follow-up literature on boys who underwent testicular tissue cryopreservation, focusing on procedural safety, testicular growth, pubertal progression, endocrine function, semen outcomes in patients reaching adulthood, and the emerging question of whether cryopreserved tissue may ultimately be needed in all cases. We will compare follow-up strategies across centers, highlight the lack of standardized endpoints, and discuss how survivorship data may refine counseling, re-consent, and future management of cryopreserved tissue. The aim is to provide a clinically oriented overview of what current longitudinal cohorts can already tell us, while identifying the key evidence gaps that still limit harmonized follow-up protocols.

## 1. Introduction

Advances in pediatric oncology have markedly improved survival, with more than 80% of children diagnosed with cancer in high-income countries now surviving for at least 5 years [1]. Cancer treatment and benign hematological diseases needing hematological stem cell transplantation (HSCT), such as sickle cell disease (SCD) and bone marrow failure, often require chemotherapy or pelvic radiation, threatening future fertility with a profound negative impact on the quality of life [2]. Since the establishment of the first formal fertility-preservation program for prepubertal boys in 2002, an international survey of 16 centers across 11 countries reported that testicular tissue had been cryopreserved from 3118 patients younger than 18 years, including patients with both malignant (60.4%) and non-malignant (39.6%) conditions [3]. Despite its growing international use, testicular tissue cryopreservation (TTC) remains experimental, as successful restoration of fertility using cryopreserved immature human testicular tissue has not yet been demonstrated [3]. In recent decades, there has been increased attention to long-term effects and consequences of chemotherapeutic agents on fertility. Alkylating agents, cisplatin, carboplatin, and radiation therapy are some of the most well-known cancer treatments involved with permanent gonadal toxicity [4,5].

Alkylating agents such as cyclophosphamide, ifosfamide, and busulfan cause DNA cross-linking, ultimately halting rapidly replicating cells and inducing their death [6]. Spermatogonial stem cells (SSCs), located within the seminiferous epithelium, are highly sensitive to DNA damage, and their integrity underpins future reproductive outcomes.

SCCs are not only highly susceptible to chemo- and radiotherapy toxic effects, but are also significantly reduced due to the presence of cancer/hematological disorders themselves [7]. In fact, prepubertal patients with cancer (solid and hematological cancer) or non-oncologic hematological disorders are more prone to present with significantly reduced spermatogonial quantity and quality compared to healthy controls, even before gonadotoxic treatments are started, leading to an urgent need to identify boys at high risk for infertility [8].

In males, preservation of Leydig-cell function may allow spontaneous pubertal onset and progression despite substantial impairment of the germinal epithelium. Consequently, normal virilization and testosterone levels do not exclude impaired spermatogenesis. Long-term follow-up should therefore separately assess pubertal progression, testicular growth, markers of seminiferous tubule and Sertoli-cell function, including FSH and inhibin B, Leydig-cell function through LH and testosterone levels, and semen parameters after puberty [9,10,11,12,13].

Despite the increasing worldwide implementation of TTC, evidence on the long-term clinical follow-up of boys undergoing this procedure remains limited. This narrative review summarizes the current evidence on long-term outcomes in prepubertal and peripubertal boys after TTC, with particular focus on procedural safety, testicular growth, pubertal development, endocrine function, semen quality after puberty, and the evolving clinical role of cryopreserved testicular tissue. We also discuss current follow-up practices, identify major knowledge gaps, and explore how survivorship data may refine fertility counseling and future clinical management of cryopreserved testicular tissue.

## 2. Materials and Methods

This narrative review was based on a literature search of English-language articles conducted in PubMed/MEDLINE and Embase from database inception to May 2026. The search combined terms related to testicular tissue preservation, patient age and pubertal status, gonadotoxic exposure, and long-term reproductive outcomes. Specifically, the following keywords and search terms were used, alone and in combination with the Boolean operators AND and OR: “testicular tissue cryopreservation”, “testicular tissue biopsy”, “prepubertal boys”, “peripubertal boys”, “gonadotoxic treatments”, “cancer”, “hematological disorders”, “long-term follow-up”, “outcomes”, “procedural safety”, “pubertal development”, “testicular growth”, “endocrine function”, “semen parameters”, and “reproductive outcomes”. We applied equivalent combinations of these terms in PubMed/MEDLINE and Embase, adapting the search syntax to each database. The search was restricted to English-language publications, with no restriction on publication year.

Original English-language, full-text clinical studies reporting follow-up data in boys or adolescents undergoing testicular tissue biopsy as part of a fertility-preservation program before or during exposure to gonadotoxic treatments were considered eligible. Studies in which testicular biopsy was performed exclusively for diagnostic purposes, without the intent of fertility preservation, were excluded.

For this review, testicular tissue biopsy (TTB) refers to the surgical procedure used to obtain testicular tissue. In contrast, testicular tissue cryopreservation (TTC) refers specifically to processing and storing the collected tissue for potential future fertility restoration. Studies reporting procedural or postoperative outcomes after TTB, as well as studies reporting long-term endocrine, pubertal, testicular, semen, or reproductive outcomes in patients whose tissue had been collected and cryopreserved for fertility preservation, were included.

Two authors independently screened the titles and abstracts of the retrieved records for eligibility. The full texts of potentially relevant articles were subsequently assessed independently by the same two reviewers according to the predefined inclusion and exclusion criteria. The reviewers resolved any disagreements about study eligibility through discussion and consensus.

Data on study characteristics and relevant clinical outcomes were extracted by one author and independently checked by a second author for accuracy and completeness. Any discrepancies were resolved by discussion and consensus.

We excluded reviews, editorials, conference abstracts without full text, non-English-language studies, studies reporting testicular biopsy performed exclusively for diagnostic purposes, and studies not reporting relevant clinical follow-up outcomes from the clinical synthesis. We also considered preclinical and translational studies when relevant to fertility-restoration strategies and future clinical translation. Studies including genetic syndromes causing reduced fertility (e.g., Klinefelter syndrome) were excluded to avoid bias in semen outcome and endocrine function.

For the section on semen and reproductive outcomes, only studies reporting postpubertal semen analyses or reproductive outcomes in patients who had undergone testicular tissue biopsy or cryopreservation were included.

## 3. Eligibility for Testicular Tissue Cryopreservation and Risk Stratification

Clinical eligibility for TTC should be assessed individually by integrating treatment-related gonadal toxicity, underlying diagnosis [8], pre-existing testicular conditions, pubertal stage, previous gonadotoxic-treatment exposure, and the patient’s medical and surgical fitness.

### 3.1. Treatment-Related Risk

To quantify the risk of adverse long-term outcomes associated with chemotherapy exposure, standardized measures such as the cyclophosphamide equivalent dose (CED) have been developed.

CED is a weighted index that converts cumulative doses of individual alkylating agents into a single cyclophosphamide-equivalent exposure, allowing comparison of overall alkylating treatment burden across different regimens [14]. According to the 2025 ESHRE Good Practice Recommendations, TTC is not recommended for boys receiving <4 g/m^2^ CED without additional gonadotoxic exposure. For cumulative exposures of 4–8 g/m^2^ CED, TTC may be considered, particularly as the cumulative dose increases, although the uncertainty regarding the risk of permanent azoospermia should be acknowledged. For exposures > 8 g/m^2^ CED, patients should be referred for fertility-preservation counseling, and TTC should be considered [13].

Myeloablative conditioning before hematopoietic stem cell transplantation (HSCT) and direct gonadal irradiation are also associated with a significant risk of infertility and represent indications for considering TTC (see Table 1 for summary recommendation). Conversely, TTC is not recommended when scattered testicular irradiation <1 Gy is the only gonadotoxic exposure [13]. Moreover, direct testicular irradiation should be distinguished from cranial or hypothalamic–pituitary irradiation, which may impair reproductive function through central endocrine dysfunction rather than through direct depletion of the spermatogonial pool [15,16,17,18,19,20].

### 3.2. Disease-Related Risk

The underlying diagnosis and previous testicular or urogenital conditions should also be considered when assessing eligibility for TTC [8,22]. Boys with severe non-oncologic hematological disorders, such as SCD and bone marrow aplasia, may already have reduced spermatogonial numbers before HSCT. Similarly, the disease itself and previous chemotherapy may reduce the number and density of spermatogonia before the planned fertility-preservation procedure, potentially affecting both the indication for TTC and its possible future utility [8,23]. A history of cryptorchidism should also be considered because it may be associated with impaired testicular function and a reduced spermatogonial pool [24].

Standard-risk acute lymphoblastic leukemia does not necessarily justify TTC at initial diagnosis, particularly when the planned treatment carries a low risk of permanent infertility [25]. However, eligibility should be reassessed if the treatment plan changes because of relapse, poor response, treatment intensification, or a subsequent indication for HSCT. Fertility counseling should therefore be considered a dynamic process, with re-referral when prognosis or treatment exposure changes [4,13].

### 3.3. Pubertal Stage and Semen Banking

Chronological age alone should not determine the fertility-preservation strategy. Semen cryopreservation is the first-line option for postpubertal adolescents who can provide a semen sample containing viable sperm. Hagenäs et al. successfully cryopreserved semen in 71 of 86 boys and adolescents. They found testicular volume to be more reliable than chronological age or reproductive hormone levels in predicting chances of retrieving viable semen. Therefore, adolescents with a testicular volume > 5 mL should be offered an attempt at semen banking before gonadotoxic treatment [26], and when viable sperm is obtained, TTC is generally not recommended [13].

Whenever possible, TTC should be performed before the initiation of gonadotoxic treatment [13]. Nevertheless, previous chemotherapy is not an absolute contraindication in an otherwise eligible patient, but fertility counseling should clarify that prior exposure, particularly to alkylating agents, may have already reduced the spermatogonial pool and therefore have decreased the expected future utility of the stored tissue.

### 3.4. Medical Fitness, Surgical Considerations, and Oncological Urgency

The final decision to perform TTC should also take into account the patient’s general condition and fitness for surgery and general anesthesia. Potential reasons not to proceed include medical instability such as high bleeding or infection risk. When feasible, testicular biopsy should be coordinated with another planned procedure requiring general anesthesia, such as central venous catheter placement or bone marrow sampling, to reduce the procedural burden [13,25].

### 3.5. Fertility-Restoration Strategies

Cryopreserved testicular tissue may be stored until adulthood; however, its future use should not be considered automatic. Before any fertility-restoration procedure, patients should undergo reassessment of spontaneous spermatogenic recovery, including semen analysis whenever feasible, evaluation of testicular and endocrine function, confirmation of their reproductive goals, and renewed consent. The original diagnosis, previous treatment exposure, quality of the stored tissue, and risk of malignant contamination should also be reviewed [27].

Potential fertility-restoration strategies include spermatogonial stem cell transplantation, autologous transplantation of intact testicular tissue fragments, and in vitro spermatogenesis using cell-based or organ-culture systems, followed by intracytoplasmic sperm injection when mature spermatozoa can be obtained [28,29]. Testicular tissue grafting has restored complete spermatogenesis in several animal models. In non-human primates, autologous grafting of cryopreserved prepubertal testicular tissue produced functional spermatozoa that resulted in healthy offspring [28]. Experimental evidence also suggests that the graft site may affect tissue survival and spermatogenic recovery, with homotopic or intratesticular grafting generally providing more favorable outcomes than ectopic or subcutaneous grafting [28,29,30].

In 2026, a first-in-human preprint reported autologous transplantation of cryopreserved immature testicular tissue in an adult who had undergone gonadotoxic treatment during childhood. Evidence of active spermatogenesis was identified in two of four intratesticular grafts, and one spermatozoon was recovered after enzymatic digestion, whereas no germ cells were identified in the subcutaneous scrotal grafts [31]. As these findings have currently been reported only in a preprint and have not resulted in a reported pregnancy or live birth, they should be regarded as preliminary proof of concept rather than definitive evidence of clinical reproductive efficacy.

The potential reintroduction of malignant cells remains another major limitation of autologous tissue or cell transplantation, particularly in patients with hematological malignancies or metastatic disease. Kourta et al. found no malignant cells using conventional hematoxylin and eosin staining, whereas disease-specific immunohistochemistry detected malignant contamination in 10 of 28 testicular tissue samples [27]. Conventional histological assessment alone may therefore be insufficient to exclude residual disease. Before transplantation, the stored tissue should undergo individualized reassessment using disease-specific immunohistochemical and, when available, molecular methods, together with multidisciplinary evaluation of the risk of malignant reseeding [27,29].

## 4. Procedural Safety

Across available pediatric TTB cohorts, the procedure was generally performed as an open unilateral testicular biopsy and was associated with low rates of predominantly minor postoperative complications. Reported short-term adverse-event rates ranged from 0% to approximately 14%, although direct comparison between studies is limited by differences in definitions, assessment methods, and follow-up duration. The most frequently reported complications were scrotal or intratesticular hematoma, postoperative pain, wound infection, transient bleeding, epididymo-orchitis, and torsion of the appendix testis [25,31,32,33,34,35,36,37,38].

In the larger contemporary cohorts, adverse events were reported in 2 of 34 patients (5.9%) by Ming et al. [39], 2 of 35 patients (5.7%) by Moussaoui et al. [40], and 3 of 139 patients (2.2%) by Kanbar et al. [34]; Barraud-Lange et al. [41] reported immediate adverse events in 3.5% of their cohort, predominantly painful episodes [34,35,36,37,38]. Most complications were self-limited or managed conservatively, without surgical revision. In the cohort of Uijldert et al., ultrasonography identified intrascrotal hematomas in 7 of 64 patients (10.9%) at 1 month, all of which had resolved by 6 months; however, small fibrotic lesions considered potentially related to the biopsy were identified in 4 of 55 patients (6.3%) at 12 months [32].

Overall, these findings support the short-term feasibility and safety of pediatric TTB. Nevertheless, the available evidence remains dominated by immediate or short-term postoperative outcomes, heterogeneous adverse-event reporting, and limited standardized long-term imaging comparing the biopsied and contralateral testes.

Current recommendations favor an open testicular biopsy, preferably performed unilaterally. Bilateral biopsy may be considered in selected cases when the expected spermatogonial pool is particularly low, but the potential benefit should be balanced against the additional surgical risk [13]. There is currently no consensus on the optimal amount of testicular tissue to retrieve. Published protocols have removed markedly different proportions, including <5% of total testicular volume, approximately one-third of one testis, and <20% of testicular tissue [34,42]. Although these approaches provided tissue suitable for cryostorage, the minimum amount required for future fertility restoration has not been established. The quantity retrieved should therefore be individualized according to testicular size, previous gonadotoxic exposure, the expected spermatogonial pool, procedural safety, and the need to retain sufficient material for future quality-control and malignant-contamination testing.

## 5. Testicular Growth and Pubertal Progression

### 5.1. Biopsied Versus Contralateral Testis

Although testicular tissue biopsy is generally considered a minimally invasive and well-tolerated procedure, local inflammation, vascular damage, and loss of testicular parenchyma could theoretically impair the subsequent growth of the biopsied testis. Uijldert et al. compared the ultrasonographic volume of the biopsied and contralateral testes at 1, 6, and 12 months after the procedure. Mean testicular volumes at these time points were 1.7 ± 2.1, 1.7 ± 2.2, and 1.9 ± 2.4 mL in the biopsied testis, compared with 1.8 ± 2.2, 1.8 ± 2.3, and 2.0 ± 2.2 mL in the contralateral testis, respectively. No significant impairment of testicular growth or clinically relevant asymmetry was identified during the first postoperative year [32]. However, a 12-month follow-up period does not allow conclusions regarding long-term testicular growth throughout puberty or into adulthood.

Borgström et al. similarly found no evident difference in size between the biopsied and contralateral testes among the seven patients who had undergone unilateral biopsy [25]. These previous findings suggest that unilateral biopsy itself does not produce marked testicular asymmetry, although the available evidence is based on small cohorts and limited long-term follow-up.

### 5.2. Overall Gonadal Growth After Gonadotoxic Therapy and TTB

The assessment of overall testicular growth must be distinguished from the comparison between the biopsied and contralateral testes, because reduced bilateral testicular volume may primarily reflect the underlying disease and subsequent gonadotoxic treatment rather than the biopsy procedure itself.

In a Belgian prospective follow-up study of 23 patients, including 15 who had undergone testicular tissue biopsy, participants were followed for a median of 4.0 years (range, 0.1–6.9 years) [37]. At the final assessment, four patients remained at Tanner genital stage G1, seven were at stages G2–G4, and twelve had reached stage G5. Testicular volume increased with pubertal progression; however, postpubertal volumes were generally reduced in both biopsied and non-biopsied patients, with a more pronounced reduction reported in the biopsied group [37]. Because biopsy exposure was closely associated with more intensive gonadotoxic treatment, these findings cannot establish a direct causal effect of the procedure itself.

Borgström et al. reported spontaneous pubertal onset or completion of pubertal development within the expected age range in all 14 participants, based on genital development and pubic-hair progression [25]. Nevertheless, testicular volume remained below the normal range in most patients. The absence of evident asymmetry between the biopsied and contralateral testes supports the assumption that reduced testicular volume was more likely related to the underlying condition and gonadotoxic treatment than to unilateral biopsy.

A Belgian two-cohort study evaluating testicular growth approximately 5 years after testicular biopsy found no significant difference in testicular volume between patients who underwent biopsy followed by gonadotoxic treatment and the comparison cohort without biopsy. Moreover, 83% of patients in the biopsy cohort had testicular volumes within the reference range for healthy controls [33]. By contrast, Delgouffe et al. reported reduced testicular volume in 9 out of 12 patients who had undergone testicular biopsy [37]. Taken together, these heterogeneous findings indicate that spontaneous pubertal progression is frequently preserved, whereas reduced overall testicular volume may still occur after gonadotoxic therapy. Current evidence does not demonstrate that unilateral biopsy impairs testicular growth, but longer follow-up with standardized bilateral ultrasonographic assessment is required.

## 6. Endocrine Function

Endocrine dysfunction, including gonadal insufficiency, is a well-known long-term side effect of gonadotoxic treatments and HSCT [42]. Given the limited clinical evidence currently available, it remains unclear whether TTB per se may also adversely affect fertility and endocrine function.

Endocrine function is assessed via multiple hormones (FSH, LH, testosterone, AMH, Inhibin B) and clinical data (testicular volume, pubertal Tanner stage).

FSH is an indirect marker of seminiferous tubule and Sertoli-cell dysfunction, whereas inhibin B provides a more direct indication of Sertoli-cell function and is associated with spermatogenic activity. FSH and Inhibin B level measurements are valuable assets for diagnosing alterations in gonadal development [43].

Inhibin B exhibits a fluctuant pattern with higher levels during mini-puberty followed by declining levels during the prepubertal stage (Tanner stage G1 has an Inhibin B median concentration of 80 ng/L). During Tanner stages G2–G3, inhibin B levels rise again. At Tanner stage G2, inhibin B is approximately 175 ng/L, whereas at G3 it is set around 215 ng/L. Its rise during pubertal development denotes functional maturation of Sertoli cells [44,45]. During adulthood, a plateau is reached with a median Inhibin B level around 188 ng/L [45,46]. As for anti-Müllerian hormone (AMH), in prepubertal boys, it reflects the presence and function of immature Sertoli cells; its concentration normally decreases during puberty as Sertoli cells mature.

LH and morning testosterone, instead, primarily reflect Leydig-cell function and should be interpreted according to age and pubertal stage as well. Since no single endocrine marker reliably establishes fertility status alone, semen analysis remains necessary after puberty whenever feasible to verify testes’ function [38].

Hormonal levels (FSH, LH, inhibin B, and testosterone) have been serially evaluated by several research groups during clinical follow-up to compare the gonadotropic profiles of patients who underwent TTB with those who did not.

Kanbar et al. evaluated FSH, LH, and testosterone concentrations after TTB and at least 2 years from completion of gonadotoxic treatment. Among 57 patients aged ≥14 years at the time of hormonal assessment, FSH was within the normal range in 36 patients (63%), whereas a total of 21 patients (37% of the whole cohort) had abnormal, either high or low, gonadotrophin levels. Elevated levels (defined as FSH ≥ 10 IU/L) were registered in 19 patients (33%), denoting primary gonadal failure, whereas low FSH levels (<1 IU/L), denoting hypogonadotropic hypogonadism, were registered in two patients (4%) [34]. The group concluded that the reproductive health outcome of those patients who underwent TTB was similar to those who did not undergo the procedure but only chemotherapy regimens, with data on hypogonadal function similar to other studies [47,48], suggesting that TTB had no direct detrimental effects on gonadal function.

Similar conclusions were drawn by Borgström et al. in their prospective longitudinal study. They reported spontaneous pubertal development and increasing testosterone concentrations (with adult levels > 10 mmol/L) in all their 14 patients who underwent TTB, none of whom required testosterone-replacement therapy [25]. However, nine patients had elevated FSH concentrations (FSH ≥ 10 IU/L), and three also had elevated LH concentrations (above the reference levels of 9.6 U/L). AMH levels were obtained for nine boys at the last visit. Two patients had normal levels according to age and pubertal stage, whereas the remaining seven had subnormal values. Inhibin B levels were measured for 8 out of 14 patients. In five boys, the levels were within the normal limit according to their laboratory reference of 5–355 ng/L for prepubertal boys and 25–325 ng/L for adults. One boy had a lower-than-normal level (Inhibin B 25 ng/L at Tanner stage 5 and 18.4 years), and two patients were below the normal range (Inhibin B 21 ng/L and < 10 ng/L for Tanner stage 5 and 4, respectively, at 18 years old). No evident differences in hormonal outcomes were identified between patients who had and had not undergone unilateral biopsy, supporting the assumption that the abnormalities were predominantly related to the underlying disease and gonadotoxic treatment rather than to the biopsy procedure itself.

A Belgian prospective cohort of 15 testicular biopsy patients sampled gonadotropic hormones throughout the years. Serum FSH and LH concentrations remained within the age- and pubertal-stage-specific reference ranges during prepuberty, and Tanner stages G2–G3 of all those patients referred to TTB [36]. Likewise, Braye et al. reported normal hormone concentrations during prepuberty and early puberty, although some abnormalities emerged in a subset of patients during mid- to late puberty. Six patients (11%) had LH concentrations > 8 IU/L, seven (13%) had FSH concentrations > 15 IU/L, and seven had testosterone concentrations < 10 nmol/L. No significant differences in FSH, LH, or testosterone and incidence of gonadal dysfunction were observed between patients who underwent biopsy and those who did not [33].

Levade and colleagues found alterations of FSH, LH, and Inhibin B in the majority (76.9%) of their patients. The significant increase in FSH concentrations (21.7 ± 7.7 IU/L) was correlated with non-obstructive azoospermia [49], similarly to results presented by the other research groups reported above. Moreover, lower levels of Inhibin B (19.1 ± 33.0 pg/ml) were observed in the azoospermic group, indicating impaired exocrine and secretory function. They also assumed that the alterations were caused more by the chemotherapy regimens than the surgical procedure itself.

Similarly, Martin et al. reported spontaneous pubertal onset in all participants, while biochemical evidence of Sertoli-cell dysfunction emerged during puberty and persisted into young adulthood. Inhibin B levels were found abnormal in 89% of patients at Tanner stage 5, along with elevated LH in 18% of patients and elevated FSH in 75%, denoting altered maturation/function of Sertoli cells; similar abnormalities persisted in adulthood [50]. See Table 2 for the summarized endocrine outcome of the above studies.

Overall, the available observational data have not demonstrated a clear additional detrimental effect of TTB on endocrine or gonadal function. However, interpretation remains limited because patients undergoing TTB subsequently received gonadotoxic treatments that are themselves well-established causes of gonadal dysfunction. Therefore, abnormalities in gonadotropins, Sertoli-cell markers, and spermatogenesis cannot be attributed independently to the biopsy procedure on the basis of the currently available evidence.

Furthermore, spontaneous pubertal progression and normal testosterone concentrations have been frequently registered with no difference between patients who underwent TTB and those who did not.

However, even though testosterone and spontaneous pubertal development appear within normal range and age, they do not exclude Sertoli-cell dysfunction or impaired spermatogenesis. Long-term surveillance is mandatory and should also include semen analyses after puberty [49,50].

## 7. Semen and Reproductive Outcomes

### 7.1. Semen Outcomes After Gonadotoxic Treatment

Postpubertal semen data from patients who underwent testicular tissue biopsy and cryopreservation remain scarce. Many patients included in the available cohort studies are still prepubertal or have not reached an age at which semen analysis is feasible. Moreover, not all eligible survivors accept or are able to provide a semen sample. Consequently, the available results are vulnerable to participation and selection bias and may not represent the entire population undergoing testicular tissue cryopreservation.

For semen nomenclature and sperm count categories according to international guidelines, see Table 3 [51].

Kanbar et al. obtained semen analyses from 27 patients who underwent TTB at a median of 6.5 years after completion of gonadotoxic treatment. Approximately 47% had normozoospermia or mild oligozoospermia, whereas 53% had severe impairment of sperm production, including azoospermia in eight patients [34]. Borgström and colleagues assessed semen parameters in six patients who had previously undergone TTB, from 4 to 9 years after HSCT; four were azoospermic, whereas the remaining two had only a small number of motile spermatozoa [25].

Delgouffe et al. reported ongoing spermatogenesis in 8 of 12 young adults evaluated after gonadotoxic treatment and to whom tissue banking had been performed: three had normozoospermia, and five had oligozoospermia, including two cases of moderate and three cases of severe oligozoospermia [37]. Levade et al. found non-obstructive azoospermia in 76.9% of the patients who agreed to provide a semen sample after completing TTB and treatment, with azoospermia associated with higher FSH and LH concentrations and lower inhibin B levels [49]. Martin et al. similarly reported that spermatozoa were detected in only a minority of postpubertal semen analyses, but patients with detectable sperm had been treated for non-malignant conditions without radiotherapy, whereas all patients with azoospermia had been treated for malignant disease [50].

See Table 4 for summarized results on semen quality.

These studies consistently point out that severe oligozoospermia or azoospermia is common after highly gonadotoxic treatment, particularly following HSCT, radiotherapy, or intensive exposure to alkylating agents. However, because testicular biopsy is performed in high-risk treatment patients, these observational data cannot establish an independent detrimental effect of testicular biopsy on spermatogenesis.

Describing a broader cohort of male childhood cancer survivors not selected according to previous TTB, Korhonen et al. provided important contextual evidence on long-term recovery and reproductive prognosis. Among 92 survivors who underwent semen analysis in adulthood, spermatogenic recovery was observed after prolonged follow-up in some patients treated exclusively with chemotherapy. By contrast, testicular irradiation ≥ 1 Gy and a CED ≥ 12 g/m^2^ were associated with an increased risk of azoospermia, while testicular irradiation ≥1 Gy and a CED ≥ 4 g/m^2^ were associated with reduced paternity rates [21]. These findings support the necessity of extended follow-up before concluding that permanent spermatogenic failure has occurred, particularly after chemotherapy-only regimens.

### 7.2. Parenthood Outcomes and Use of Cryopreserved Tissue

Data on natural conception, paternity, use of assisted reproductive technologies, donor sperm, and other routes to parenthood have not been systematically reported in cohorts of boys who underwent TTC. Moreover, no peer-reviewed pregnancy or live birth resulting from the clinical use of cryopreserved immature human testicular tissue has been reported yet. The first-in-human transplantation findings reported in 2026 remain preliminary, have not demonstrated reproductive efficacy, and should be considered separately from established clinical outcomes.

Semen outcomes after treatment must therefore be distinguished from outcomes resulting from the use of stored testicular tissue. At present, the available human follow-up studies primarily describe spontaneous residual or recovered spermatogenesis after gonadotoxic therapy, but they do not demonstrate fertility restoration attributable to the cryopreserved tissue. Long-term follow-up should document semen parameters, attempts at natural conception, paternity, use and outcomes of assisted reproduction, use of donor sperm, and whether the cryopreserved tissue was retained, discarded, transferred, or used experimentally.

## 8. Discussion and Clinical Considerations

Available follow-up data suggest that TTB is technically feasible and generally associated with minor and self-limited short-term complications. Comparisons between the biopsied and contralateral testes have not demonstrated a consistent biopsy-related impairment of testicular growth. However, these findings derive from relatively small and heterogeneous cohorts, with limited follow-up into adulthood and few untreated or appropriately matched control groups.

Spontaneous pubertal onset and testosterone are frequently preserved after childhood gonadotoxic treatment and TTB. Nevertheless, reduced bilateral testicular volume, elevated FSH, low inhibin B, and impaired semen parameters are observed in a substantial proportion of survivors, particularly following HSCT, radiotherapy, or intensive alkylating-agent exposure but with no increased incidence in those patients who had previously undergone TTB [21,25,33,34,36,37,38,52,53].

Normal virilization and testosterone concentrations should not therefore be interpreted as evidence of preserved spermatogenesis. A complete hormonal assessment (FSH, LH, testosterone, inhibin B) is mandatory, including pubertal progression evaluation. Moreover, bilateral testicular volume during clinical follow-up and semen analysis should always be performed after puberty whenever feasible. An adult testicular volume ≥ 15 mL could be predictive of non-azoospermia, even though definitive data can be assessed only with sperm analysis [21]. The registered semen alterations usually found in TTB patients are consistent with the expected gonadal damage caused by high-risk treatment [47,48] and do not provide evidence of an additional biopsy-related effect; however, the studies are still too scarce to analyze the effect of biopsy from treatment and disease-related factors.

The available evidence should be interpreted cautiously. Most follow-up studies include relatively small observational cohorts, frequently from single centers, with heterogeneous underlying diseases, gonadotoxic exposures, and follow-up durations. Appropriate control groups are uncommon, and only a proportion of patients have reached adulthood or undergone semen analysis. These limitations restrict direct comparisons between studies and make it difficult to isolate the potential effects of testicular tissue biopsy from those of the underlying disease and subsequent gonadotoxic treatment.

At present, TTC should be considered only for carefully selected prepubertal or early pubertal patients who cannot provide viable sperm and who face a clinically significant risk of permanent infertility. The procedure should be undertaken within an experienced fertility-preservation program, with appropriate ethical oversight and explicit counseling regarding its experimental nature [13]. For postpubertal adolescents able to provide a semen sample, sperm cryopreservation remains the preferred option.

Furthermore, fertility counseling should be dynamic rather than limited to the initial diagnosis [49]. Whenever possible, TTC should be performed before gonadotoxic therapy; however, previous treatment is not an absolute contraindication in an otherwise eligible patient, but families should be informed that previous alkylating-agent exposure may reduce the potential future utility of the stored tissue [13]. Moreover, eligibility to TTC should be reassessed after relapse, poor response, treatment intensification, or a subsequent indication for HSCT. Whenever feasible, biopsy should be coordinated with another clinically indicated procedure requiring general anesthesia, provided that this does not delay oncological treatment.

Long-term care should include transition from pediatric survivorship services to adult andrology or reproductive-medicine follow-up. Follow-up should also investigate reproductive desire, attempts at natural conception, information about alternative fertility-preservation options, paternity outcomes, and whether the stored tissue is retained, transferred, discarded, or considered for experimental use.

It is also important to consider that a small subgroup of patients could encounter delayed recovery of spermatogenesis several years after chemotherapy-only regimens, whereas persistent impairment is more likely present following direct testicular irradiation or radiation-containing HSCT conditioning [21]. Therefore, spontaneous recovery should be taken into consideration and always assessed before any future fertility intervention involving the stored tissue.

## 9. Future Directions and Clinical Translation

Future research should determine which survivors will actually require their cryopreserved tissue and which will experience spontaneous spermatogenic recovery.

Priorities include the development of validated criteria for selecting patients for tissue use, optimization of graft site, fragment size [34,41,54] and transplantation timing, reliable exclusion of malignant contamination, and demonstration of the functional and genetic competence of spermatozoa obtained from cryopreserved tissue.

A first-in-human report published as a preprint in 2026 described histological evidence of spermatogenesis after autologous transplantation of cryopreserved immature testicular tissue with changes in the semen parameters during follow-up. Although this represents an important proof of concept, it remains preliminary and has not yet demonstrated a peer-reviewed pregnancy or live birth [55]. Clinical application should therefore remain restricted to carefully regulated research settings with multidisciplinary assessment and renewed informed consent.

Pharmacological gonadoprotection also remains investigational. As of this moment, no drug-based intervention has an established clinical role in protecting spermatogonial stem cells in boys undergoing gonadotoxic treatment, and hormonal suppression or other experimental agents should not replace established fertility-preservation approaches [56]. Future preclinical and clinical studies may identify effective gonadoprotective strategies, but their safety must be demonstrated without reducing the efficacy of oncological treatment.

## 10. Conclusions

The current evidence is still limited. Small sample sizes, single-center or single-program designs, heterogeneous underlying disease inclusions and treatment exposures, and substantial variation in follow-up duration are the main limitations.

As of this moment, TTC and TTB appear feasible and acceptably safe for selected prepubertal and early pubertal boys facing high-risk gonadotoxic therapy, but heterogeneity regarding the volume of tissue retrieved, and surgical and cryopreservation protocols is still an important limitation, and longer follow-up is still needed.

Furthermore, study protocols vary in hormone panels analyzed during follow-up, methods of testicular-volume measurement, definitions of endocrine dysfunction, and semen analysis.

Standardized endpoints and predefined assessment time points are required to allow meaningful comparison between centers. International registries should collect procedural complications, testicular growth measurements, pubertal and endocrine outcomes, and semen parameters. Moreover, they are fundamental for collecting reproductive outcomes, long-term management, and possible use of stored tissue.

In conclusion, multicenter-design studies are necessary to overcome the current paucity of data and heterogeneous follow-up. We suggest incorporating endocrinology evaluations at standardized time points across all clinical centers. Annual clinical follow-up could be offered to all prepubertal boys. Instead, given the greater vulnerability to gonadotoxic effects during peripubertal and pubertal stages [34], clinical evaluation could be tightened to every 6 months for patients with Tanner stage > 1. Tanner stage assessment, including testicular volume, height, and weight, should be measured at every endocrinologic appointment, while gonadal levels could be assessed once a year. Moreover, Inhibin B should be evaluated at completion of pubertal development [44,45].

It would be very useful to offer sperm collection at 16 years old, and if sufficient and viable sperm are obtained, cryopreservation could follow. Once patients enter adulthood, we also believe they should be referred to an andrologist [49] for continued follow-up.

**Figure 1:**
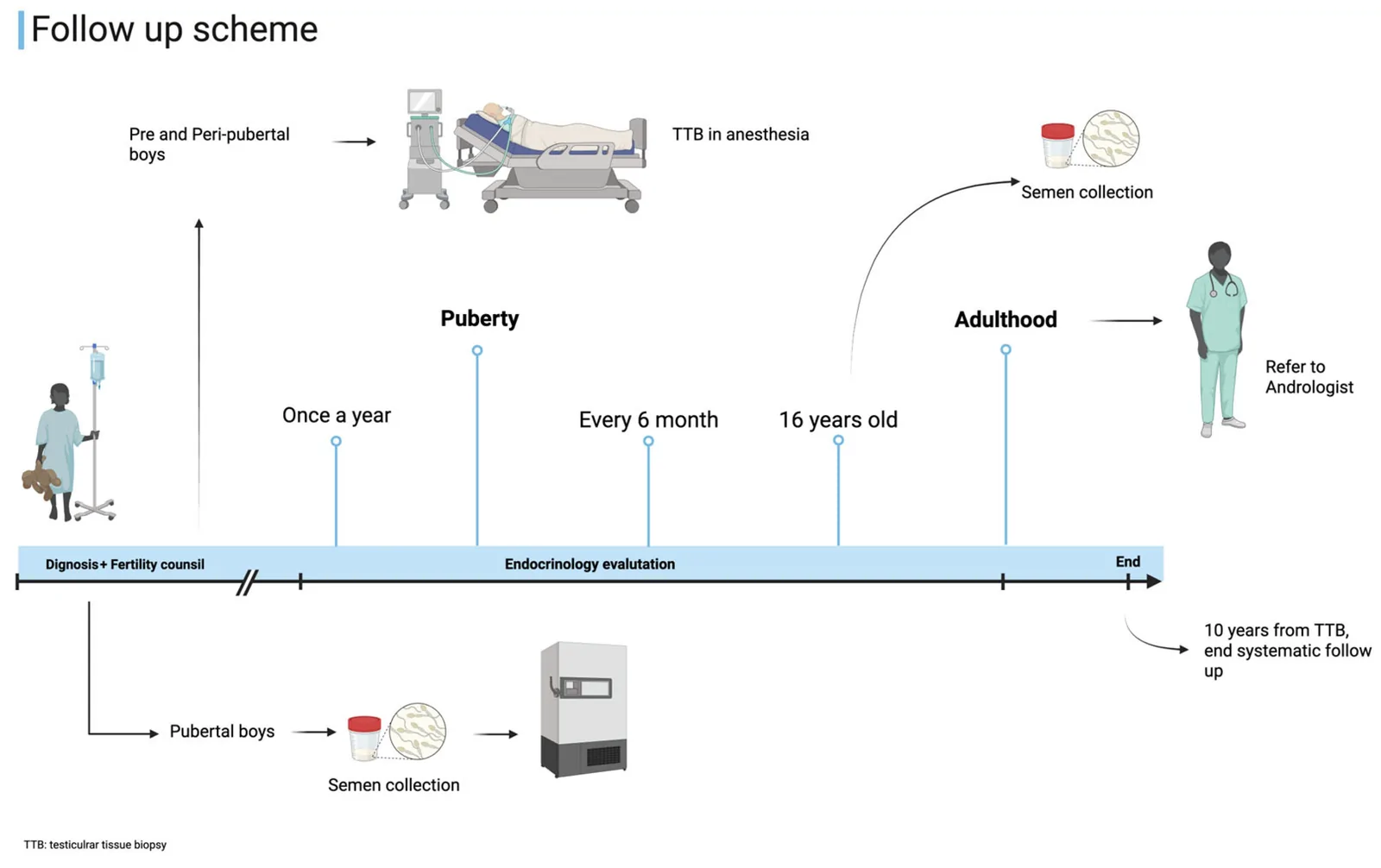
A representative figure of a possible follow-up timeline, from diagnosis until adulthood.

An extended follow-up period, such as ten years after TTB and gonadotoxic treatment, would be very important. In fact, a repetition of sperm collection before termination of long-term follow-up could detect those patients undergoing delayed spermatogenic recovery [21], enhancing future fertility chances. Moreover, regular follow-up years after termination of gonadotoxic treatment would be crucial to provide support to fertility-preservation programs and guidance for future fertility restoration options (see Figure 1).

At the time of diagnosis, pubertal boys should undergo semen collection and cryopreservation prior to the initiation of therapy. Whereas, in prepubertal and peripubertal boys, testicular tissue cryopreservation should be considered for fertility preservation. Endocrinological assessment should be performed annually until the onset of puberty and subsequently every six months until the age of 16 years. At this stage, semen collection should be offered, including to patients who have previously undergone testicular tissue cryopreservation. Upon reaching adulthood referral to an andrologist could be very important for individualized assessment and management of reproductive and fertility-related issues.

## Figures and Tables

**Table 1:** Recommendation summary.

Therapy	Fertility Outcome
Gonadotoxic treatment < 4 g/m^2^ CED with no other associated gonadotoxic treatment	Low infertility risk
Gonadotoxic treatment between 4 and 8 g/m^2^ CED	Consider TTC, but paucity of scientific data on long-term detrimental effects on gonadal function at this dosage
Gonadotoxic treatment > 8 g/m^2^ CED	Suggested TTC **
Hematopoietic stem cell transplant conditioning regiment, including TBI	Suggested TTC
Testicular radiotherapy > 6 Gy	Suggested TTC

CED: Cyclophosphamide equivalent dose; TTC: testicular tissue cryopreservation; TBI: total body irradiation; ** Even though TTB is highly suggested, it has been described as a possible recovery of spermatogenesis in long-term follow-up of patients treated exclusively with chemotherapy [21].

**Table 2:** Summary of the main endocrinological outcome.

	Cohort	Mean Follow-Up Duration	Endocrine Outcome
Borgström et al. 2020 [25]	14 boys (aged 1.5–14.5 years) who underwent TTB prior to HSCT	7.2 years after HSCT	All underwent spontaneous pubertal development and normal increase in testosterone with adult levels > 10 mmol/L. However, 9 had elevated FSH (FSH ≥ 10 IU/L) and 3 reported also high (>9.6 U/L) LH concentrations. Out of 9 AMH-tested patients, only 2 had normal levels.
Kanbar et al. 2020 [34]	57 patients underwent TTB and gonadotoxic treatments, all aged ≥14 years at time of hormonal assessment	At least 2 years after completing gonadotoxic treatment	FSH was normal in 63% of patients. 33% had elevated levels (FSH ≥ 10 IU/L), indicating primary gonadal failure, while 3% had low FSH indicating hypogonadotropic hypogonadism.
Delgouffe et al. 2026 [36]	15 patients underwent TTB and gonadotoxic treatment, median age 3.6 (neonatal—14.9). Twenty patients had a malignant disease and three non-malignant.	4.0 (0.1–6.9) years	Throughout the follow-up after TTB, all patients had FSH and LH concentrations remaining within the age and pubertal-stage ranges. However, 11% had higher LH concentrations and 13% had higher FSH concentrations during mid-late puberty.
Levade et al. 2024 [49]	31 patients underwent TTB and chemotherapy	7.4 (2–14) years	Alterations of either FSH, LH or Inhibin B levels were found in 76.9% of patients.
Martin et al. 2026 [50]	50 prepubertal boys underwent TTB before gonadotoxic treatments. 52% had malignant hematological disorders, 34% non-malignant diseases, 14% a solid tumor. 80% underwent HSCT after TTB.	Not reported	All patients experienced spontaneous pubertal onset, but at complete pubertal development, 18% had elevated LH, 75% elevated FSH, and 89% low inhibin B, denoting Sertoli-cell dysfunction.

TTB: testicular tissue biopsy; HSCT: hematopoietic stem cell transplantation.

**Table 3:** Semen nomenclature and sperm count categories according to international guidelines.

Nomenclature	Definition
Azoospermia	Absence of spermatozoa
Oligozoospermia	Sperm less than 15 million/mL
Normospermia	Sperm ≥ 16 million/mL

**Table 4:** Summary of semen outcome.

Author	N Patients	Median Years from Therapy Stop	Semen Results
Kanbar et al. 2020 [34]	27	6.5	47% normospermia; 53% had severe impairment of sperm production (including 8 patients with azoospermia)
Borgström et al. 2020 [25]	6	4–9	4 patients azoospermia; 2 few motile spermatozoa
Delgouffe et al. 2023 [37]	12	12.3	3 normozoospermia, 5 had oligozoospermia (including 2 cases of moderate and 3 cases of severe oligozoospermia)
Levade et al. 2024 [49]	36	2–14	76.9% non-obstructive azoospermia
Martin et al. 2026 [50]	50	Unspecified	12 patients provided with semen, 42% had spermatozoa

## Data Availability

These data were derived from the following resources available in the public domain: PubMed/MEDLINE [https://pubmed.ncbi.nlm.nih.gov].

## Abbreviations

The following abbreviations are used in this manuscript:

TTB	Testicular tissue biopsy
TTC	Testicular tissue cryopreservation
SSCs	Spermatogonial stem cells
SCD	Sickle cell disease
HSCT	Hematological stem cell transplantation
CED	Cyclophosphamide equivalent dose
TBI	Total body irradiation
